# A Case of Brachial Plexus Injury Induced by Intraoperative Positioning during Thoracoscopic Resection of a Mediastinal Tumor

**DOI:** 10.70352/scrj.cr.25-0680

**Published:** 2026-03-27

**Authors:** Daiki Takei, Takuro Miyazaki, Ryoichiro Doi, Koichiro Shimoyama, Daisuke Taniguchi, Tomohiro Obata, Satoshi Mizoguchi, Soichiro Kiya, Keitaro Matsumoto

**Affiliations:** Department of Surgical Oncology, Nagasaki University, Nagasaki, Nagasaki, Japan

**Keywords:** brachial plexus injury, mediastinal tumor, intraoperative positioning, thoracoscopic surgery

## Abstract

**INTRODUCTION:**

Brachial plexus injury is a rare but serious complication of thoracic surgery performed in the lateral position. Improper positioning of the upper limb or neck can increase the risk of nerve damage. Risk factors include excessive abduction of the upper limb and hyperextension of the neck. Preventive measures—such as preoperative simulation of surgical positioning, regular intraoperative reassessment of limb and neck alignment, and close collaboration among surgeons, anesthesiologists, and nursing staff—are critical for ensuring patient safety.

**CASE PRESENTATION:**

A 40-year-old female diagnosed with an anterior mediastinal neuroendocrine tumor underwent thoracoscopic resection in the left lateral position. During intraoperative repositioning, fixation of the right upper limb became dislodged, resulting in external rotation of the arm and neck extension. Postoperatively, she developed right upper limb weakness and sensory disturbances consistent with brachial plexus injury. MRI revealed swelling and high signal intensity of the brachial plexus on short tau inversion recovery imaging. Conservative treatment with oral mecobalamin and rehabilitation was initiated. Her symptoms gradually improved, and full recovery was achieved by POD 60.

**CONCLUSIONS:**

Brachial plexus injury, although rare, is a potentially serious complication of surgery that may cause long-term sequelae. Greater awareness of position-related nerve injury is essential for safer thoracic surgical practice and improved postoperative outcomes.

## INTRODUCTION

Thoracic surgery is performed in the lateral decubitus position; the affected-side upper limb is usually elevated and secured, and intraoperative changes in patient positioning may be necessary. During such repositioning, brachial plexus injury can occur as a result of the limb or neck position. While some cases resolve spontaneously, others may lead to significant and lasting neurological deficits.

Here, we report a case of brachial plexus injury that developed during thoracoscopic resection of an anterior mediastinal tumor performed in the left lateral decubitus position.

## CASE PRESENTATION

**Fig. 1:**
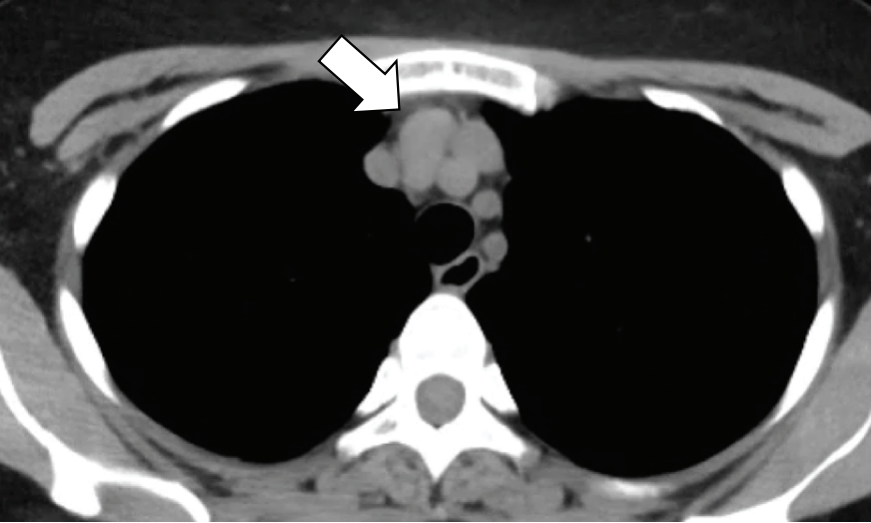
Chest CT showing a 2.5 × 1.5-cm nodule (white arrow) located between the right brachiocephalic vein and brachiocephalic artery in the upper mediastinum.

The patient was a 40-year-old female (height 161 cm, weight 60.8 kg) with a medical history of hypothyroidism and right mastopathy. During a routine health screening, a chest X-ray revealed an abnormal shadow. Chest CT showed a 2.5 × 1.5-cm nodular lesion located between the right brachiocephalic vein and brachiocephalic artery in the upper mediastinum (**Fig. 1**). Pathological examination by endoscopic ultrasound-guided fine-needle aspiration, performed by the gastroenterology department, confirmed the diagnosis of a neuroendocrine tumor, and surgical resection was planned.

On admission, physical examination and laboratory studies, including pulmonary function testing, electrocardiography, and blood chemistry, revealed no significant abnormalities. Tumor marker levels were within normal limits.

Surgery was performed in the left lateral decubitus position under 1-lung ventilation. The first port was placed in the right thorax at the fifth intercostal space along the mid-axillary line for thoracoscopic observation. The procedure was conducted under artificial pneumothorax with carbon dioxide insufflation. Three additional ports were inserted in the right thorax: at the third intercostal space along the anterior axillary line, the third intercostal space along the posterior axillary line, and the fourth intercostal space along the anterior axillary line. The mediastinal pleura was incised to expose the bilateral brachiocephalic veins. Dissection proceeded cranially, and the tumor was identified between the bilateral brachiocephalic veins and the right brachiocephalic artery.

**Fig. 2:**
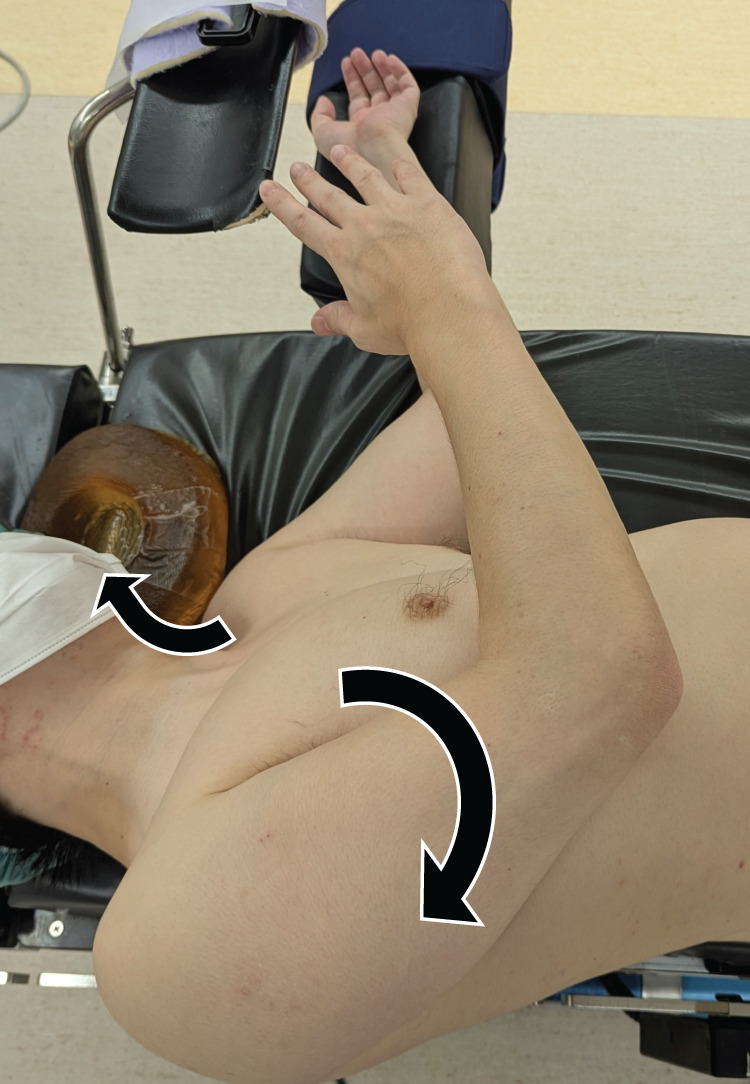
Reconstruction of the intraoperative positioning by the author. External rotation of the right upper limb and extension of the neck are demonstrated (black arrow).

At 33 min into the surgery, the operating table was rotated to the right to improve the surgical field. After 62 min, dislodgement of the right upper limb fixation was noted. Although the position was promptly corrected, external rotation of the arm and neck extension had already occurred (**Fig. 2**). The tumor was resected and submitted for frozen section analysis, which suggested Castleman disease. The operation was therefore concluded with tumor excision only. The total operative time was 238 min, and intraoperative blood loss was 10 ml.

**Fig. 3:**
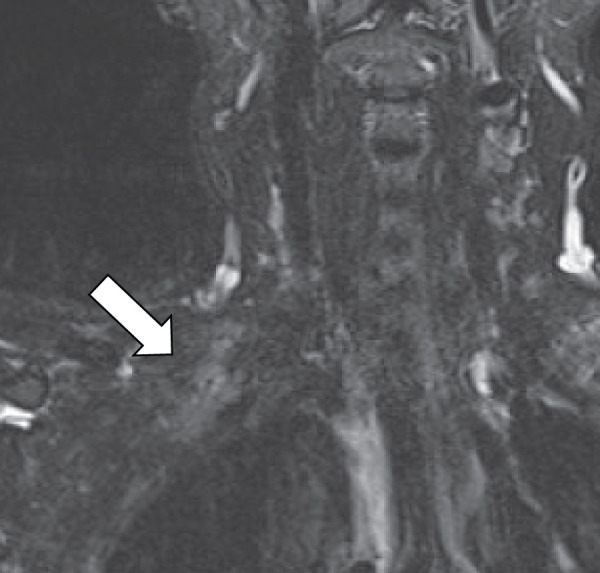
Cervical MRI showing swelling and high signal intensity (white arrow) of the brachial plexus on short tau inversion recovery imaging at the dorsal side of the central right clavicle, consistent with brachial plexus injury.

Immediately upon awakening from anesthesia, the patient showed incomplete paralysis of the right shoulder and upper arm. Sensory examination revealed decreased superficial sensation in the C5 dermatome, and manual muscle testing demonstrated muscle strength of 1–2 in the same region. Muscle strength in the C6 distribution was also reduced to approximately grade 4. MRI showed swelling and high signal intensity of the brachial plexus in the dorsal region of the right subclavian area on short tau inversion recovery imaging (**Fig. 3**). No other organic abnormalities were identified, and the radiological findings corresponded with the patient’s neurological symptoms. The brachial plexus injury was therefore attributed to intraoperative external rotation of the upper arm and neck extension following dislodgement of the limb fixation.

Oral mecobalamin therapy and rehabilitation were initiated. Aside from the neurological symptoms, the postoperative course was uneventful, and the patient was discharged on POD 5 with ongoing outpatient rehabilitation. By POD 30, the strength of the right upper extremity had improved to grade 3 on manual muscle testing, and by day 60, both motor and sensory symptoms had nearly completely resolved, with only minimal residual neurological deficit.

## DISCUSSION

Brachial plexus injury associated with surgery has been reported at a frequency of 0.02%–0.06%, making it a rare complication.^1)^ The main causes are considered to be intraoperative positioning and direct surgical manipulation of the nerves, with mechanisms including mechanical compression, overstretching, and autoimmune inflammatory responses.^2–4)^ In addition, Ben-David and Stahl^1)^ identified excessive abduction (>90°), external rotation of the upper limb, and excessive head rotation as risk factors for brachial plexus injury. However, no standardized treatment strategy has been established.^5)^ While most cases resolve without sequelae, complete symptom resolution has been reported to take a median of 20 weeks (range, 8–50 weeks).^6)^

Only 7 cases of brachial plexus injury associated with thoracic surgery in the lateral decubitus position have been reported, including the present case (**Table 1**).^5,7–11)^ All of these involved upper-type injuries, suggesting that traction caused by excessive abduction of the upper limb may predispose patients to brachial plexus injury. In addition, thoracic outlet syndrome was proposed as a risk factor in these reports, and preoperative provocative testing in the 90° abduction–external rotation position (Wright test), which assesses compression of the subclavian artery in the costoclavicular space, was recommended.^5,7,8)^ Although vascular compromise due to physiological narrowing does not directly cause brachial plexus injury, compression of the brachial plexus can occur at the same site as pulse obliteration. It has therefore been suggested that thoracic outlet syndrome may lower the threshold pressure required to induce nerve injury.^8)^

**Table 1:** Reported cases of postoperative brachial plexopathy following thoracic surgery in the lateral decubitus position in Japan

Reference	Sex	Age (years)	Primary disease	Surgical position	Upper limb position	Operative time (min)	Involved roots	Initial treatment	Treatment duration (days)
Ohata et al.^7)^	M	34	Giant bullous emphysema	Right lateral position	120° abduction	288	C5–Th1	Methylprednisolone	12
Sugibe et al.^5)^	M	20s (reported)	Mediastinal tumor	Right semi-lateral position	NA	430	C5–Th1	Mecobalamin	180
Tahara et al.^8)^	M	35	Pyoderma	Right lateral position	120° abduction	417	C5, C6	Prednisolone	168
Shimoe et al.^9)^	M	12	Right upper lobe bronchial atresia	Left lateral position	120° abduction	330	C5–Th1	Prednisolone	28
Go et al.^10)^	M	52	Bronchopleural fistula	Left lateral position	90° abduction	360	C5–Th1	Prednisolone	194
Ozaki et al.^11)^	F	45	Mitral regurgitation	Left semi-lateral position	90° abduction	NA	C5, C6	NA	90
Present case	F	38	Mediastinal tumor	Left lateral position	90°external rotation	178	C5, C6	Mecobalamin	61

F, female; M, male; NA, not available

In this case, the brachial plexus injury was likely caused by external rotation of the upper limb and extension of the neck that occurred when upper limb fixation became dislodged intraoperatively. Although rotation of the operating table was anticipated, preoperative simulation of patient positioning was not performed because difficulty in securing the surgical field had not been expected. The absence of such simulation may therefore have contributed to the injury through loss of fixation and subsequent neck extension. As preventive measures, we have since implemented mandatory preoperative simulation of surgical positioning in any case where intraoperative repositioning might be required, along with postoperative verification of positioning by nursing staff after table rotation and more frequent pressure relief.

To avoid such complications during thoracic surgery in the lateral decubitus position, it is essential to perform a thorough preoperative physical assessment; adopt intraoperative positioning that avoids excessive abduction (>90°), external rotation of the upper limb, and excessive head rotation; and ensure secure fixation of the patient to the operating table to prevent positional shifts. Regular intraoperative checks by anesthesiologists and nursing staff are also critical. Brachial plexus injury has also been reported in the supine position under conditions such as excessive upper limb abduction or excessive head rotation, underscoring the need for constant vigilance regarding patient positioning during surgery.^12–14)^

## CONCLUSIONS

Brachial plexus injury caused by surgical positioning is a serious complication that can significantly impair QOL if symptoms persist. Risk factors include compression of the nerve roots and excessive stretching due to upper limb abduction beyond 90° and external rotation of the arm. In cases where intraoperative positional changes are anticipated, preventive measures such as preoperative positioning simulations are essential to ensure patient safety and reduce the risk of nerve injury.
